# Preparation of Monolithic Capillary Chromatographic Columns Using Supercritical Fluid as a Porogen Solvent

**DOI:** 10.1007/s10337-014-2651-7

**Published:** 2014-03-16

**Authors:** Michał Szumski, Bogusław Buszewski

**Affiliations:** Faculty of Chemistry, Department of Environmental Chemistry and Bioanalytics, Nicolaus Copernicus University, Gagarina 7, 87-100 Toruń, Poland

**Keywords:** Capillary liquid chromatography, Monolithic polymeric column, Porogen solvent, Supercritical carbon dioxide

## Abstract

Monolithic polymeric beds were synthesized in fused silica capillaries using either trimethylolpropane trimethacrylate (TRIM) or a mixture of butyl methacrylate (BMA) with ethylene glycol dimethacrylate (EDMA) as monomers. Carbon dioxide at temperature and pressure conditions above its critical values was used as a porogen solvent. The purpose of using the supercritical carbon dioxide was to have the possibility of changing the solvation power (and thus the porosity of the resulting monolith) of the porogen by pressure and temperature changes instead of changing the porogen composition. The experiments were performed using a special setup consisting of a stainless steel high-pressure reactor to which the fused silica capillary was connected. The synthesized monoliths underwent liquid chromatographic evaluation. The polyTRIM capillary monoliths were characterized by different permeability, which depended on the pressure of the synthesis. BMA/EDMA columns were applied for separation of alkylbenzenes and a model mixture of proteins.

**Electronic supplementary material:**

The online version of this article (doi:10.1007/s10337-014-2651-7) contains supplementary material, which is available to authorized users.

## Introduction

During last two decades, monolithic macroporous materials have become very popular in analytical chemistry and particularly in separation science because of several advantages they provide. First of all, monoliths let the chromatographist overcome serious problems related to preparation of packed capillary columns as well as their often unpredictable stability and lifetime. Secondly, monoliths can be synthesized in different formats—from classical columns through capillaries to channels of chip devices. Thirdly, in comparison to particulate-based stationary phases beds, they provide much higher mass transfer [1–5].

A serious advantage of the monolithic supports over particulate materials is their easy (in general) way of preparation which relies on a chemical process (polymerization or polycondensation) induced in a liquid solution, which is introduced into a tube or a chip channel [6]. There have been elaborated several types of monolithic stationary phases, namely silica, polymer-based and those of mixed inorganic–organic nature [7–11].

Silica monoliths have been elaborated as counterparts of classical particulate silica gel stationary phases, and the most significant input in this field was presented by the group of Tanaka [12–18]. They are synthesized from alkoxysilanes in a series of hydrolysis and condensation reactions in a presence of an aqueous polymer (PEG) solution as a porogen solvent. Fine pore tuning can be done by flushing with ammonium hydroxide solution or using ammonium obtained from thermal decomposition of urea, which is added to the polymerization mixture. Synthesis of silica monolithic columns is a multistep process which results in a highly (in comparison to particulate-based bed) permeable stationary phase which can be subsequently chemically modified to obtain desired surface properties [19].

Preparation of polymeric monolithic columns is, in general, easier than those silica-based. Typically, it is a single-step synthesis in which the monophasic homogeneous mixture of the monomers (functional and cross-linking) and a porogen solvent are polymerized to form a porous uniform “rod” in a mold (tube, capillary or chip channel). Usually, the polymerization is triggered by a thermal or photochemical decomposition of an initiator. A tuning of the properties of polymeric monolithic stationary phase can be done by:

- monomer/cross-linker ratio,
- amount of the initiator,
- temperature of the process (UV and thermal polymerization),
- irradiation intensity and time (UV polymerization),
- porogen quality (single solvent or multicomponent porogen) and quantity (monomers/porogen ratio),
- time of the polymerization,
- grafting.

Another class of monoliths, which behave like reversed-phase liquid chromatographic stationary phases are photopolymerized sol–gel monoliths prepared from 3-(trimethoxysilyl) propyl methacrylate (*γ*-MAPS) using both sol–gel and polymerization processes [20, 21].Very interesting and less popular approach to synthesis of organic monoliths via ring opening metathesis polymerization (ROMP) was proposed by Buchmeiser et al. [22, 23].

It is believed that a key parameter in preparation of monolithic columns is to use a proper porogen solvent. A porogen, which is a single solvent or a mixture, should provide a complete miscibility of the constituents of the polymerization mixture and should yield a desired porosity of the monolith. Such a requirement is especially difficult to be fulfilled when molecularly imprinted polymers are to be synthesized in a single-step synthesis, because of the presence of the template to be imprinted, monomers of different chemical characters and the initiators.

A porogen solvent should be therefore a good solvent for the monomers, but for the polymer rather weak one. However, its solvation power has the influence on the phase separation moment, which in turn may affect the porosity of the polymer. Some of the multicomponent porogens allow for fine tuning of the monolith morphology by simply changing the ratios of its constituents. From this point of view, supercritical fluid can play a role of an ideal single component porogen, the solvation power of which can be adjusted by the change in their density.

Supercritical fluid (SF) is defined as a compound being under temperature and pressure conditions higher than its critical values. SFs have very useful properties, such as: changeable density, low viscosity and surface tension while values of diffusion coefficients are between those observed in gasses and liquids. These properties make them very useful in extraction, chromatography and chemical synthesis including polymerization [24–28]. One of the most frequently used SF is carbon dioxide which is characterized by low critical values, it is safe, cheap and is also regarded as a “green” solvent. CO_2_ cannot be oxidized, it is miscible with many monomers and fluoroorganic compounds, it is also neutral during free radical reactions, and being a gas under ambient conditions, it can be removed from the reaction environment just by decompression of the system.

There have been presented several examples of application of supercritical CO_2_ in preparation of various materials [29] including polyurethane foams [30–32], microspheres [33–35], aerogels [36] and polymeric monoliths [37–39].

Preparation of polymeric monoliths was reported by Cooper et al. [39] who polymerized trimethylolpropane trimethacrylate (TRIM), ethylene glycol dimethacrylate (EDMA) and trimethylolpropane trimethacrylate/methacrylic acid (TRIM/MAA) polymers using CO_2_ above its critical conditions. They used special high-pressure vessels equipped with sapphire windows, which allowed for observation of solubilization and phase separation processes. In another work, they focused on influence of pressure on polymer porosity. The authors noticed that the pressure changed during the polymerization process and reported the dependence of the porosity of the final observed pressure [38]. They also reported that a high content of the monomer (40–60 %) resulted in the formation of the monolith, which conformed to the internal dimensions of the reaction vessel, while lower monomer content resulted in the formation of microspheres [39].

Although, as it was described above, the synthesis of a monolith using CO_2_ in a relatively large mold was possible, to our knowledge there was no literature report on doing so in tubes of smaller diameter including capillaries. Hence, the main objective of this work was to assess possibility of synthesis and evaluate the factors (including technical ones) influencing the preparation of polymeric porous monoliths in a capillary format using carbon dioxide above its critical conditions as a tunable and “green” porogen solvent.

## Materials and Methods

### Materials and Chemicals

Fused silica capillaries of 100 and 25 μm internal diameter and 375 μm outer diameter (TSP100375 and TSP025375, respectively) were purchased from Composite Metal Services (Ilkley, Worcester, UK).

Trimethylolpropane trimethacrylate (TRIM), butyl methacrylate (BMA), EDMA and azobisisobutyronitrile (AIBN, were purchased from Sigma Aldrich Chemie GmbH (Steinheim, Germany). 3-(Trimethoxysilyl) propyl methacrylate (γ-MAPS) was from Fluka (Buchs, Switzerland). Sodium hydroxide, methanol, acetone, toluene and thiourea (all analytical grade) were purchased from Polskie Odczynniki Chemiczne (POCh, Gliwice, Poland). Acetonitrile (HPLC grade) was from J.T. Baker (Witko, Łódź, Poland). Deionized water was produced in our laboratory using Milli-Q (Millipore, Bedford, MA, USA) water purification system. Carbon dioxide (purity 4.6) was purchased from BOC (Mysłowice, Poland).

### Instrumentation

**Fig. 1:**
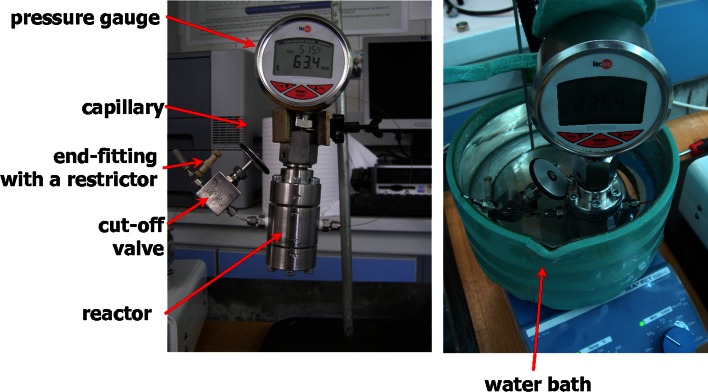
Setup for synthesis of monoliths in capillaries using SC-CO_2_ as a porogen

The SF polymerization was carried out in a home-made high-pressure vessel (a reactor) to which the fused silica capillary was connected (Fig. 1). The vessel consists of a stainless steel thick-walled cylinder, both sides of which were closed with removable caps equipped with Teflon seals. The caps are kept in place by six screws each. Upper cap was equipped with a digital manometer connected through a chemical separator. The chemical separator is a part having a steel membrane which is in contact with a medium being measured and transfers (via a non-compressible internal liquid) a pressure to the manometer. Thus, the separator protects the manometer itself from the direct contact with the medium (in our case—a polymerization mixture). Two holes were drilled and tapped on the opposite sides of the reactor cylinder. Through one of these holes, CO_2_ was introduced into the reactor via a cutoff valve, while to the second hole the capillary was connected. The volume of the reactor was determined to be 14.0 mL. CO_2_ was delivered using a constant pressure air-driven pump of the supercritical fluid extractor SE-1 (Seko, Brno, Czech Republic), which is capable of providing pressure up to 40 MPa. To complete a polymerization process, the reactor together with the capillary was immersed in a water bath.

The chromatographic measurements were performed using a capillary LC setup consisting of Rheos 2000 pump (Flux Instruments, Reinach, Switzerland), two-position ten-port Cheminert C72MX valve with a microelectric actuator and a 50 nL injection loop (Valco, Houston, TX, USA) and UV–Vis Spectra-100 detector (Thermo Separation Products, San Jose, CA, USA). The Clarity AD converter and software (DataApex, Prague, Czech Republic) were used for data collection and control of the system. A simple splitter (T-piece with a fused silica capillary) was used between the pump and the valve, which allowed to run the pump at higher flow rate and thus to reduce a gradient delay. A pressure versus flow relationship (according to the procedure described in the ref. [40]) was measured using an air-driven constant pressure HPLC pump from Knauer (Knauer GmbH, Berlin, Germany). The SEM micrographs were taken using Leo 1430 VP apparatus (Leo Electronenmikroskopie, GmbH, Oberkochen, Germany).

### Preparation of the Columns

The fused silica capillaries were etched and silanized with γ-MAPS according to the procedure mentioned earlier [40]. The objective of this step was to covalently bind the polymer to the capillary inner surface and thus to increase the mechanical stability of the bed and avoid the extrusion of it from the capillary during the chromatographic process. The top cap of the reactor and the cutoff valve were mounted to the main cylinder, and the construction was turned upside down. Then, the mixture of monomers containing the initiator was introduced, and a small magnetic bar was added. The vessel was closed with the bottom cap, and the entire reactor was turned to a horizontal position, that is, in such a way that the side hole with union for the capillary was pointing upward, and the side hole with the cutoff valve pointed downward. Then, through the cutoff valve, the gaseous CO_2_ was gently passed through the polymerization mixture for 15 min. After that the capillary was connected to the vessel using a piece of PEEK tubing (1/16″ outer diameter and 0.5 mm inner diameter) and a PEEK ferrule. The other end of the capillary was closed using either of the two systems:

(a) A PEEK union using a finger-tight PEEK ferrule and 1/32″ PEEK sleeve on one side and the finger-tight plug on the other side of the union.

(b) A flow restricting device consisting of a PEEK union used in system (a) to which a short piece (10 cm) of 25 μm i.d. fused silica capillary was connected which outlet was closed using the same type of union and a plug as in system (a). The unions, ferrules and plugs we used were from Upchurch (Chromatographie Handel Müller GmbH, Fridolfing, Germany).

The vessel with the connected capillary was then placed on the magnetic stirrer, the SFE pump was connected to the cutoff valve, and the CO_2_ was introduced to the reactor until a desired initial pressure was achieved. Keeping the reactor still on the stirrer, it was gently heated with a stream of warm air until the pressure of ca. 13 MPa was achieved. Then, the capillary end fitting was gently open for a few seconds to introduce the polymerization mixture into the capillary. The reactor together with the capillary was submerged in the water bath and left for 20 h to complete the polymerization at 60 °C. After that it was cooled down to the ambient temperature and the screws retaining the upper and bottom caps were gently released to allow system to depressurize slowly. The capillary was disconnected from the vessel, flushed with methanol and subjected to the evaluation of the hydrodynamic properties.

## Results and Discussion

The constructed reactor was found to be a safe and easy to operate device in which monoliths can be synthesized. According to the reports of Cooper et al. [37–39], such monomers or their mixtures as TRIM, EDMA and TRIM/MAA dissolve easily in SC-CO_2_ (above a specific pressure) and can polymerize to form a monolith if the monomer concentration is higher than 40 %. During the initial experiments, we used either TRIM or the BMA/EDMA mixture as the monomers (Table 1). Both systems resulted in porous polymers which (in most cases) filled entire space of the reactor and could be removed from it as one piece. The samples of monoliths taken from different places (bottom, middle and top part of the vessel) were observed with SEM to assess their structural differences. We did not observe any differences in the morphology of the polymers if only the initial pressure exceeded 10.3 MPa at 60 °C (denoted as *P*_i_^60 °C^–polymerization temperature). For such conditions, we noticed that the polymerization mixture did not fill the reactor homogeneously—the porous polymer filled only the lower part of the vessel, leaving the upper space partially empty while the structure of some parts of the polymer were characterized by different morphology. This observation is to some extent consistent with the data of Cooper et al. [37–39] who chose minimum polymerization pressure of *P*_i_^60 °C^ 12–13 MPa as the value of the lowest pressure limiting the miscibility of the monomers (TRIM) with CO_2_. The slightly higher initial pressure of *P*_i_^25 °C^ = 6.5 MPa allowed us to achieve higher pressure of 24.5 MPa at 60^ °C^, which provided full miscibility.

**Table 1:** Preparation conditions of monolithic BMA columns

Polymer	% Mon	*P*_i_^25 °C^ (MPa)	*P*_*i*_^60 °C^ (MPa)	*P*_f_^60 °C^ (MPa)	*P*_f_^25 °C^ (MPa)	Δ*P* ^60 °C^ (MPa)	Δ*P* ^25 °C^ (MPa)	*S*_BET_ (m^2^ g^−1^)	Median pore diameter (nm)	Pore volume (mL g^−1^)
1	50	8.0	27.88	22.88	6.75	−5.0	−1.25	19.5	n.d.	n.d.
2	40	8.0	28.04	24.64	7.04	−3.4	−9.6	n.d.	n.d.	n.d.
3	50	10.0	32.87	22.63	6.45	−10.24	−3.55	28.3	n.d.	n.d.
4	45	8.0	30.72	24.38	6.13	−6.34	−1.87	1.7	n.d.	n.d.
5	45	10.0	37.35	27.04	7.32	−10.31	−2.68	15.5	381	1.50
6	45	10.0	36.55	26.50	6.62	−10.05	−3.38	11.1	466	1.53
7	45	10.0	38.45	27.67	8.68	−10.78	−1.32	5.5	633	1.69
8	45	10.0	37.00	26.90	8.33	−10.10	−1.67	11.9	716	1.58
9	40	8.0	31.17	25.85	7.92	−5.32	−0.08	1.18	12,725	1.57
10	45	6.5	30.39	24.50	7.68	−5.89	1.18	4.8	1,685	1.55
11	45	5.0	10.32	13.20	6.26	2.88	1.26	6.2	770^a^	1.08
12	45	10.0	37.49	37.29	8.79	−0.20	−1.21	–	–	–

In all polymers, 2 % of AIBN added, except polymer no. 7—1.5 % if initiator, 8—1 % of initiator; 9—the same conditions as 2 but initial pressure was adjusted using method (ii)—see “Results and Discussion” for details; 12—no initiator added

*P*_i_ *, P*_f_ initial and final pressure at 25 or 60 °C

^a^ Note that polymer 11 did not fill entire volume of the reactor

In the next step, the attempt to synthesize the monoliths in fused silica capillaries was made. As it was described in the experimental section, the capillary was directly connected to the pressurized polymerization vessel in which the homogeneous polymerization mixture was produced. Capillary was then filled with the mixture by gentle opening its outlet for 3–4 s. We used two types of end fittings—without and with the restrictor. We noticed that filling the capillary without the restrictor was quite difficult—the process was very fast, and sometimes it was difficult to close the end fitting plug (because of the high pressure) which led to losses of the polymerization mixture or it could led to lack of a tight seal of the end fitting. It was also easy to observe that very fast pressure drop in the capillary occasionally caused a kind of foaming of the mixture in the capillary which left visible bubbles in the channel, and, as a result-free spaces in the polymer.

The application of the restrictor capillary (connected to the free end of the 100 μm i.d. capillary column) of 25 μm i.d. restricted the flow of the polymerization mixture significantly which allowed for filling the column more slowly (thus avoid foaming) and, on the other hand, let to close the outlet more easily. We found that the restrictor length of ca. 10 cm was enough to provide a proper flow.

**Fig. 2:**
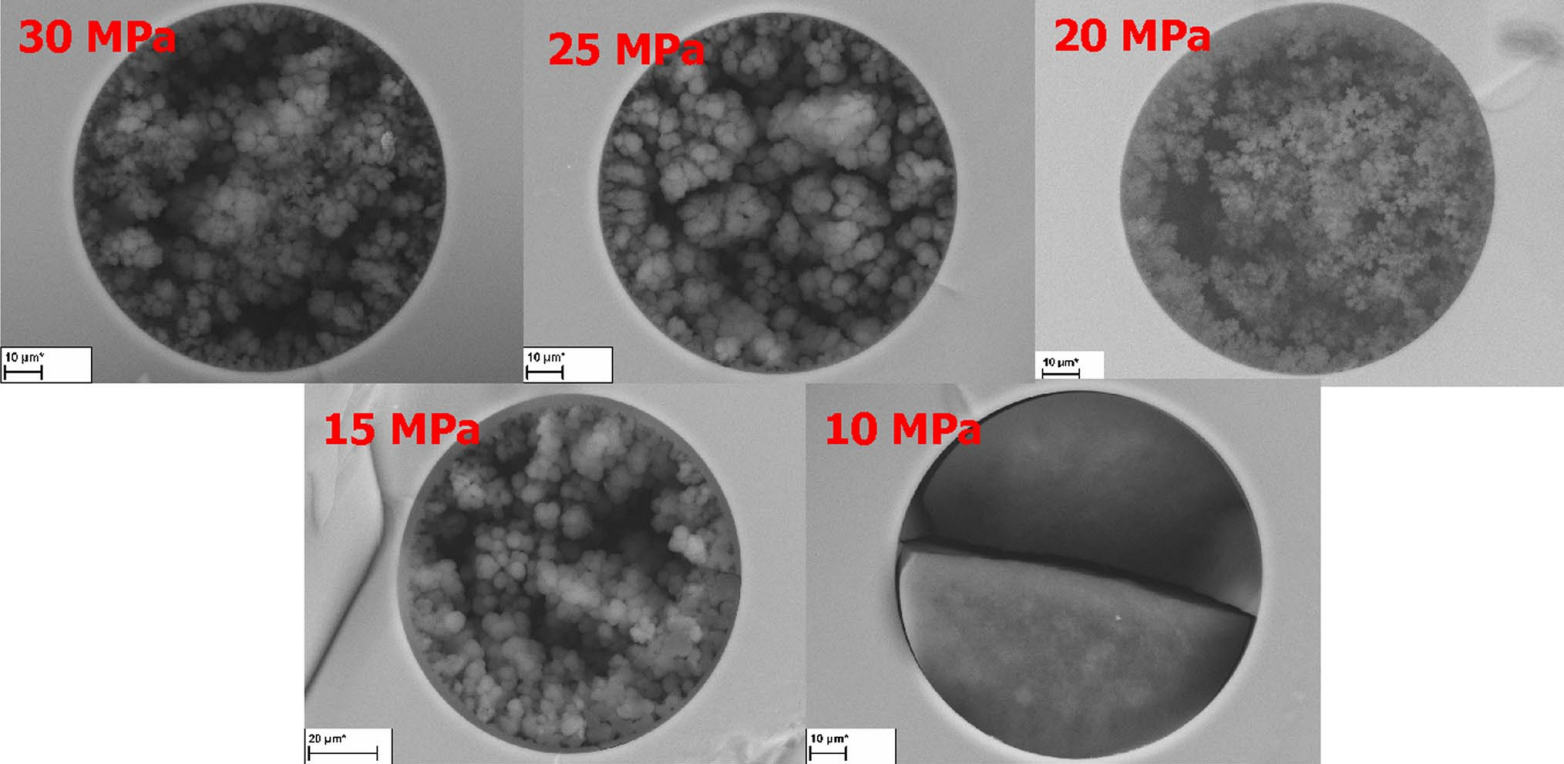
SEM pictures of polyTRIM synthesized under constant pressure conditions at 60 °C

It is known from the work of Hebb et al. [38] that significant pressure changes can be observed during polymerization process using SC-CO_2_ as a porogen. At the initial steps of our experiments, we attempted to produce monolithic TRIM columns under constant pressure conditions, without the inlet cutoff valve, so the system could be called “open.” We were able to do so, because the pressure could be then controlled (increased and released when necessary) by the SF extractor air-driven pump. Despite the fact that the TRIM columns did not provide any chromatographic properties (in the terms of being able to separate), they showed some differences in hydrodynamics (see supporting information). The SEM pictures of these columns are presented in Fig. 2. It is shown in Fig. 2 that the bed prepared at 10 MPa was not porous at all, and in consequence was completely impermeable. The permeability of the monoliths prepared at 15 and 20 MPa did not practically differ from each other at all and were very low (see supporting information). However, increasing the pressure to 25–30 MPa caused a substantial change in the monoliths structure making them much more permeable. It was quite surprising, however, that the SEM picture of the column prepared at 15 MPa was not consistent with its flow-pressure plot, and the globules were bigger than in the monolith prepared at 20 MPa. The possible explanation of this phenomenon can be supported by the observations of Hebb et al. [38] who reported that pore size and surface area are highly sensitive to the changes of monomer concentration in the system in the pressure range of 15–18 MPa, where monomers did not form fully homogeneous mixture with SC-CO_2_.

We also tried to use the “open system” to synthesize BMA/EDMA columns but without any success. The formed monolithic columns were not mechanically stable, and the bed could be partially removed from the capillary during flushing with methanol and then leaving porous fragments at the inner capillary wall.

We finally decided to give up using the “open system” because we observed that the some polymeric viscous material could be found as far as in the switching valve placed close to the pump (they were transported there either by diffusion or rather because of pressure adjustments), which made the SFE system exposed to the risk of permanent contamination.

A final polymerization system was a “closed system” equipped with an inlet cutoff valve, which is the same approach as it is found in the literature. First problem was to determine how the initial pressure is to be set. In general, it can be solved in two ways:

1. The monomers with initiator are placed in the reactor and after the mixture is sparged with gaseous CO_2_, the chamber is filled with carbon dioxide to the desired pressure, then the inlet valve is closed. After that the reactor is placed for ca. 10 min on the magnetic stirrer and gently heated to homogenize the polymerization mixture initially, followed by immersing it in the water bath placed on the magnetic stirrer with a heating plate.
2. In the second approach, the procedure is generally the same but at the beginning, after the initial pressure is reached, the components are stirred for ca. 2 min. Then, because a slight pressure drop is usually observed (CO_2_ is dissolved in monomers, but still there exist two phases) more CO_2_ is added until the desired *P*_i_^25 °C^ is reached again. These steps are repeated as long as pressure drops after stirring. Further procedure is the same as procedure 1.

The differences between the above procedures may have a noticeable effect on the initial, maximum and final pressure at 60 °C (different amounts of CO_2_ are introduced). So, we decided to follow the second approach as more accurate, as the mixture is fully saturated with CO_2_.

The “closed system” was applied for synthesis of BMA/EDMA monoliths. One can notice that in comparison to TRIM monoliths, the BMA/EDMA polymers are characterized by much finer structures and taking into account their rather low-specific surface areas (*S*_BET_–see Table 1), they do not have extensively developed internal porous structure.

**Fig. 3:**
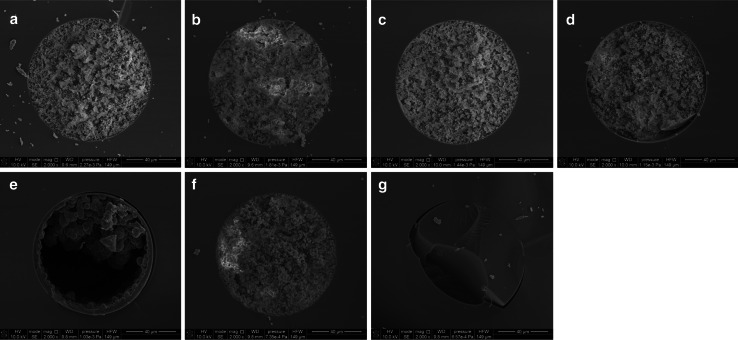
SEM images of the cross sections of selected monolithic BMA/EDMA columns. The column numbers are compatible with the labels in the Table 1. **a** column 5, **b** column 6, **c** column 7, **d** column 8, **e** column 9, **f** column 10 and **g** column 11

The morphology of the BMA/EDMA polymers synthesized in the capillaries differed to some extent from the bulk material in the reactor. Unfortunately, in many cases, free spaces were visible in the cross section of the capillary. As shown in Fig. 3e, the void in the capillary is on one side of the capillary cross section, while globular polymer is on the other side, which may suggest that some gravity phenomena could have played a role during formation of the polymer. In our opinion, such observation may result from two groups of effects. On one hand, it is very likely, that the “confinement effects” described by He et al. [41] may be the source of the differences between rather homogeneous bulk polymer and the capillary monolith. On the other hand, these phenomena can be probably connected with simultaneous influence of two other effects: (i) because of its low viscosity supercritical medium is not able to “hold” the creating polymer and (ii) the density and solubility of the supercritical porogen were locally very low which in consequence resulted in the creation of polymer very similar to that of no. 11 (Fig. 3g), which was synthesized under the low initial pressure. These data strongly suggest that it is very difficult to predict whether the same pressure-solubility-concentration conditions will be maintained over entire length of the capillary during synthesis of the polymeric monolith in the capillary column of 20–30 cm length. Because of the specific dimensions of the capillary (they are of small diameter and relatively long), it is very likely that if the polymerization starts in one place in the capillary the created porous polymer can block (not completely of course, because it is porous) the fragment of the capillary and in this way to restrict the pressure adjustments in other parts of the capillary. In this way, the local conditions in some parts of the capillary may be different from those in the reactor, and, if the polymer itself has small pores (like BMA/EDMA in comparison to TRIM) such effects can be even stronger. The SC carbon dioxide is probably too strong solvent for the BMA/EDMA system resulting in small pores, however, decreasing the reaction pressure in order to decrease the solvation power of the porogen may result in decrease in solubility of monomer mixture. On the one hand, lower concentrations of the monomers (40 %–polymer 9) and lower pressure (*P*_i_^25 °C^ = 8.0 MPa) result in a globular monolith in the reactor, however, in the capillary the globular structures can also be observed but in the vicinity of large voids (Fig. 3e). On the other hand, synthesis of the polymer using 50 % of monomers gave completely impermeable capillary columns. We decided to use 45 % of monomers, and those monoliths occupied entire cross section of the capillaries, but still were characterized by very small pores. Here, the reproducibility of the monoliths synthesized using SF porogen can be considered from two points of view. On the one hand, the characteristics of bulk materials prepared without the connected capillary (so the systems were closed throughout all the process) were more or less consistent. For example, median pore diameters of two monoliths prepared in the same way as monolith 5 equaled 352 and 330 nm. But on the other hand, the monoliths 5 and 6 polymerized with the capillaries connected differed much more—381 and 466 nm, respectively. So, it is very likely that filling of the capillary may give some effect as small portion of the monomers (and CO_2_ as well) is removed from the reactor. We believe that the accuracy of the filling step seems to be crucial, taking into account such a discrepancies in pore diameters. The differences in the mentioned capillaries 5 and 6 (they were synthesized to check the reproducibility) are hard to explain—the SEM pictures and observations did not revealed any structural differences in their cross section. However, the observations of the capillary 6 made under the optical microscope have shown the presence of many small cracks perpendicular to the capillary channel. The segments of the monolith (each was 5–10 mm long) must have had some non-porous fragments as the column was not permeable.

**Fig. 4:**
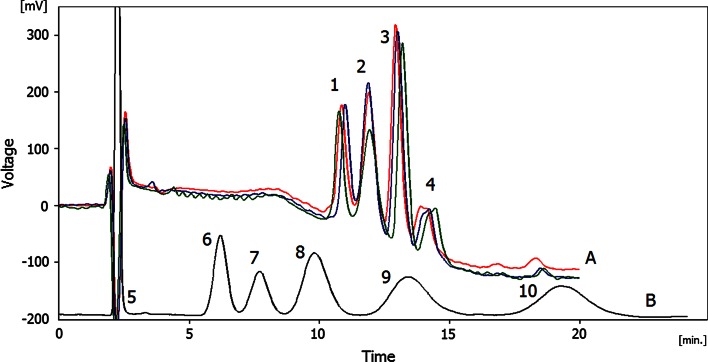
Exemplary separations performed on column 5. Column length *L* = 15 cm, *F* = 0.5 μL min^−1^. *a* Separation of the mixture of proteins (resolved peaks: *1* ribonuclease, *2* cytochrome c, *3* myoglobin, *4* ovalbumin). Chromatographic conditions: mobile phase A: 0.1 % of formic acid in water, mobile phase B: 0.1 % of formic acid in acetonitrile. Gradient conditions: A for 1 min, then 80 % B in 10 min and 80 % B for 5 min. Detection at *λ* = 214 nm; *b* separation of alkylbenzenes (peaks in order of elution: *5* thiourea, *6* benzene, *7* toluene, *8* ethylbenzene, *9* propylbenzene and 10 butylbenzene). Mobile phase: 55/45 ACN/H_2_O, detection UV at *λ* = 200 nm

Only two columns, 5 and 7, after being cut to the length of 15 and 16 cm, respectively, showed some chromatographic behavior. Both columns exhibited rather poor performance for low molecular weight analytes (low number of the theoretical plates as calculated for benzene, however, it must be mentioned that the asymmetry factors for alkylbenzenes were of good values—between 1.0 for benzene and 1.3 for butylbenzene) and, as shown in Fig. 4, column 5 worked better during the separation of four proteins. As it was demonstrated in the Fig. 4 the reproducibility of the separation of the tested protein mixture was good. The small differences between the chromatograms we attributed to not reproducible gradient profile of our LC pump which operated at relatively low flow rates.

The Kozeny–Carman relationships allowed us to calculate the pore and mean particle sizes of these two columns :

1 $$ d_{\text{pore}} = 2(5B/\varepsilon_{\text{T}} )^{0.5} $$

where *d*_pore_ is pore diameter (μm), *B* is permeability (m^2^) and *ε*_T_ is total porosity.

Permeability *B* can be calculated from the following equation:

2 $$ B = \eta Lu/{\varDelta}P $$

where *η* is mobile phase viscosity (Pa s), *L* is column length (cm), *u* is linear velocity of the mobile phase (cm min^−1^) and Δ*P* is pressure drop across the column (Pa).

Porosity *ε*_T_ was calculated from:

3 $$ \varepsilon_{\text{T}} = V_{0} /V_{\text{C}} = \, Ft_{ 0} /V_{\text{C}} $$

where *V*_0_ is void volume of the column (μL), *V*_C_ is volume of the empty tube (μL) and *t*_0_ is elution time of unretained compound (*t*_0_ marker) (min).

Mean particle size, *d*_*p*_, was also calculated for these columns:

4 $$ d_{\text{p}} = \, (1{-}\varepsilon_{\text{T}} )(180B/\varepsilon_{\text{T}} ^3)^{0.5} $$

In the Table 2, the selected parameters of these two columns with our previously presented monolithic stationary phase based on photopolymerized BMA–EDMA material were compared [40]. The monoliths synthesized using the SF-CO_2_ as a porogen were characterized by worse efficiency; however, their selectivities (benzene/toluene) were slightly higher. The columns 5 and 7 were also characterized by a little bit higher hydrophobicity, which suggests slightly different surface properties. The calculated pore sizes for column 5 and 7 were 0.47 and 0.59 μm, respectively, and the particle sizes equaled 0.92 and 0.94 μm, respectively. The photopolymerized monolith was characterized by larger pore and particle diameters, which made it more permeable and efficient [40]. It is clear that the calculated pore and particle diameters of SF monoliths are too low to obtain a good liquid chromatographic performance, particularly in the terms of the column permeability. Changing initiator content (2, 1.5 and 1 %) in the polymerization mixture (polymers 5, 7 and 8) did not result in any significant differences in columns’ hydrodynamics, nor any differences in SEM picture was observed. However, the median pore diameter, measured in the bulk polymer samples (see Table 1), increased with decreasing the initiator content, which was typical effect described in the literature. One can also notice that median pore diameters are a bit smaller than those calculated from the column permeability.

**Table 2:** Comparison of parameters of columns 5 and 7 as well as one of the previously described photopolymerized BMA/EDMA monolithic column [40]

	Monolith 5	Monolith 7	Photopolymerized BMA/EDMA
*L* (cm)	15	16	9.8
*d*_pore_ (μm)	0.47	0.59	1.29
*d*_p_ (μm)	0.92	0.94	1.34
*N*_*T*_ max (plates/m)	15,323	21,942	47,574
*R*_toluene/benzene_	1.4	1.30	2.34
*α*_toluene/benzene_	1.9	1.87	1.72
Hydrophobicity	log *k* = 0.1578*n*_*c*_ + 0.2326	log *k* = 0.1451*n*_*c*_ + 0.1628	log *k* = 0.1251*n*_*c*_ + 0.044

## Final Remarks

It is hard to formulate any general conclusions regarding preparation of polymeric monoliths in fused silica capillaries using supercritical CO_2_ as a porogen solvent. First of all, it must be stated that from the technical point of view the method seems to be more complicated (compared with the traditional methods which are based on liquid porogen solvent) because of necessity of using high-pressure system. Although the idea of using “green” porogen characterized by adjustable solvent strength seems to be perfect, it looks that the pressure and solubility conditions in a capillary format are not so easy to be controlled. Moreover, it must be emphasized that for BMA/EDMA system, SF-CO_2_ turned out to be too strong solvent to obtain desirable pore sizes. Decrease in the pressure was not a good solution as very low pressure should be employed, which could cause problems with initial solubility, and the result was the voids in the monolith. Also, it is noteworthy that filling of the capillary seems to be crucial to obtain a monolith without voids, which can be visible in a capillary channel. The restrictor method described in the work seems to be relatively simple and efficient to do that, however, not ideal one. The bulk polymer samples revealed different surface areas, which could not be directly connected with the polymerization conditions, and the BMA/EDMA polymers did not show globular morphology similar to polyTRIM materials.

## Electronic Supplementary Material

Below is the link to the electronic supplementary material.

Supplementary material 1 (PDF 392 kb)
