# Supplementary material for: Preparation of Monolithic Capillary Chromatographic Columns Using Supercritical Fluid as a Porogen Solvent
Source: Chromatographia. 2014 Mar 16;77(15):1009–17. doi: 10.1007/s10337-014-2651-7 (PMC4111859; doi:10.1007/s10337-014-2651-7)
Supplement: Supplementary file 1 — Supplementary material 1 (PDF 392 kb) [file 10337_2014_2651_MOESM1_ESM.pdf]

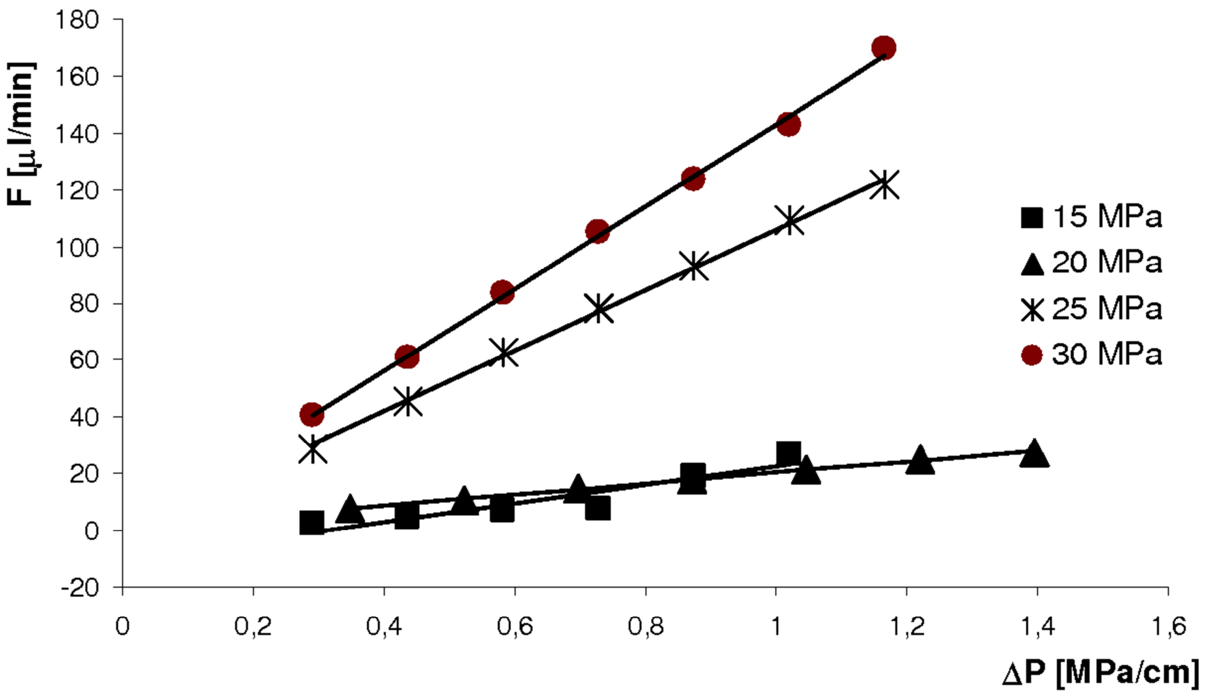

Fig. 5. Comparison of the permeability of the polyTRIM monolithic capillary columns synthesized under constant pressure conditions.
